# Hypoparathyroidism-retardation-dysmorphism syndrome—Clinical insights from a large longitudinal cohort in a single medical center

**DOI:** 10.3389/fped.2022.916679

**Published:** 2022-07-22

**Authors:** Odeya David, Rotem Agur, Rosa Novoa, David Shaki, Dganit Walker, Lior Carmon, Marina Eskin-Schwartz, Ohad S. Birk, Galina Ling, Ruth Schreiber, Neta Loewenthal, Alon Haim, Eli Hershkovitz

**Affiliations:** ^1^Pediatric Endocrinology Unit, Saban Pediatric Medical Center for Israel, Soroka University Medical Center, Beer Sheva, Israel; ^2^Faculty of Health Sciences, Ben-Gurion University of the Negev, Beer Sheva, Israel; ^3^Diagnostic Radiology Department, Soroka University Medical Center, Beer Sheva, Israel; ^4^Morris Kahn Laboratory of Human Genetics, National Center for Rare Diseases, Faculty of Health Sciences, National Institute for Biotechnology in the Negev, Genetics Institute at Soroka University Medical Center, Ben-Gurion University of the Negev, Beer Sheva, Israel; ^5^Pediatric Gastroenterology Unit, Saban Pediatric Medical Center for Israel, Soroka University Medical Center, Beer Sheva, Israel; ^6^Pediatric Nephrology Unit, Soroka University Medical Center, Beer Sheva, Israel

**Keywords:** HRD, Sanjad-Sakati, infections, seizures, nephrolithiasis, bowel obstruction

## Abstract

**Background:**

Hypoparathyroidism, retardation, and dysmorphism (HRD) Syndrome is a rare disease composed of hypoparathyroidism, retardation of both growth and development, and distinctive dysmorphic features. Here, we describe the long-term morbidity and mortality in a large cohort of HRD patients and suggest recommendations for follow up and treatment.

**Methods:**

Medical records of 63 HRD syndrome patients who were followed at Soroka Medical Center during 1989–2019 were reviewed retrospectively. Information regarding demographics, medical complications, laboratory findings, and imaging studies was collected.

**Results:**

The mortality rate was 52%. The main causes of death were infectious diseases including pneumonia, septic shock, and meningitis. Multiple comorbidities were found including brain anomalies in 90% of examined patients (basal ganglia calcifications, tightening of corpus callosum, Chiari malformation, hydrocephalous, and brain atrophy), seizures in 62%, nephrocalcinosis and/or nephrolithiasis in 47%, multiple eye anomalies were recorded in 40%, bowel obstructions in 9.5%, and variable expression of both conductive and senso-neural hearing loss was documented in 9.5%.

**Conclusion:**

HRD is a severe multisystem disease. Active surveillance is indicated to prevent and treat complications associated with this rare syndrome.

## Introduction

Hypoparathyroidism, retardation, and dysmorphism (HRD) syndrome, also termed Sanjad–Sakati syndrome (1–5), is an autosomal recessive syndrome exhibiting hypoparathyroidism, both intra-uterine and post-natal growth retardation, developmental delay, and typical dysmorphic features (OMIM # 241410). It is caused by mutations mapped to chromosome 1q42-q43, encoding Tubulin-specific chaperone E (TBCE) (3). This protein product is important for microtubule assembly pathways. Other features of HRD include immunodeficiency with hyposplenism and impaired neutrophil function (6), ocular anomalies including microphthalmia, refractory errors, corneal opacities, and nystagmus (7), as well as impaired dentation with extensive carries and cryptorchidism. Recently, multiple other endocrinopathies have been recognized as being associated with HRD including hypothyroidism, cortisol deficiency, hypoglycemia, and hypogonadism (8). To date, the high mortality rate was attributed to infectious causes (6). Treatment is mostly supportive and includes calcium supplements and active vitamin D metabolites, prophylactic antibiotic treatment, anti-pneumococcal vaccines, and hydrocortisone and L-thyroxin as needed.

The common cause for HRD among the Arab population in the Middle East is homozygosity for the TBCE (NM_003193.4) founder mutation c.155_166delGCCACGAAGGG (p.Ser52_Gly55del) (3). Previously reported prevalence of HRD in Kuwait was 7–18 per 100,000 live births (9). The estimated prevalence in our hospital is 10 per 100,000 live births. Due to the high consanguinity rate among the Bedouin population of southern Israel, that constitute ~50% of the births in our medical center, the estimated incidence is about 1 per 5,000 live births within this group.

In this manuscript, we describe the multiple comorbidities associated with HRD, including neurological, gastrointestinal, and renal manifestations in our cohort and in previously reported cases, and recommend treatment guidelines.

## Methods

This is a population-based retrospective cohort analysis. All patients with HRD diagnosed clinically and later through molecular genetic testing at Soroka University Medical Center (our center is the only medical center in the Negev area of southern Israel) were included in the current study. Recruitment was based upon diagnoses in medical records of patients who visited our center during 1989–2019. Patients with hypoparathyroidism due to other causes and those with incomplete medical records were excluded from the study. Data were collected using patient records from the hospital's computerized and manual databases.

Collected demographic data included: age, sex, date of birth, date of death, and cause of death. Morbidity data included diagnoses made by primary care physicians, pediatric endocrinologists, and during hospitalizations. Number and causes for ward and ICU (intensive care unit) admissions were recorded. Laboratory data included blood count, electrolytes, urea, creatinine, and bacterial cultures. Imaging data details included ultrasonography (US), computed tomography (CT), and magnetic resonance imaging (MRI). All CT scans and MRIs from 1995 on were reviewed by a single neuroradiologist. Encephalogram (EEG) and electromyography (EMG) results were recorded. The data in this study were processed and analyzed with IBM SPSS software, version 23. The study was approved by the local ethics committee of Soroka University Medical Center.

## Results

### Demographics

Sixty-three patients (38 female/25 male), mean age 6.2 years (range: birth to 29 years) were followed in our center during 1989–2019. Forty-seven (74%) were diagnosed at birth, and 15 (24%) were diagnosed at a mean age of 53 days (range 14–320 days). Genetic diagnosis was available in 25/63 (40%) of patients. Except for a single patient homozygous for a novel *TBCE* c.207-208delTA mutation, all patients diagnosed genetically were homozygous for the common c.155_166delGCCACGAAGGG mutation. No unique findings were related to the novel TBCE mutation. Chorionic villus sampling was made in one patient (2%). Thirty-three patients (14 males/19 females) had died by December 2019, constituting a mortality rate of 52% (Supplementary Table 1). Causes of death were mainly infectious diseases, including pneumonia, septic shock, and meningitis. One patient died of ventricular arrhythmia. Amoxicillin prophylaxis was begun during the year 2000, followed by vaccination with conjugated pneumococcal vaccine. Twenty-two patients were included in this cohort from 1989–2000, and of them, 17 died before the age of 5 years (77%). Forty-one patients were included in this cohort from 2001–2019, and of them, 10 died before the age of 5 (24%) (*p-*value 0.013). The median age of death for all cohort patients was 2 years and 4 months (range 2 months−29 years).

### Hospital admissions

Sixty-three patients had a total of 775 hospitalizations, with an incidence of 2.01 per life year. The five most common causes for admissions were hypocalcemia (45%), fever (33%), seizures (25%), dehydration (13%), and hypoglycemia (12%); note, that some patients had more than one cause of admission. Pediatric intensive care unit admissions represented 9% of all hospital admissions (Table 1). Most hospitalizations occurred during early infancy, with an incidence of 4.65 per life years ranging from birth to the second year of life and declining slowly to 1.19 per life years in patients older than 20 years.

**Table 1:** Hospitalization characteristics of HRD patients in Soroka University Medical Center.

	**Incidence**	**Incidence per life years**
Total hospitalizations (*n*)	775	2.01
ICU hospitalizations (*n*, %)	71, 9%	0.18
Hypocalcemia—associated admissions (*n*, %)	350, 45%	1.21
Fever—associated admissions (*n*, %)	256, 33%	0.66
Seizures—associated admissions (*n*, %)	194, 25%	0.50
Dehydration—associated admissions (*n*, %)	104, 13%	0.27
Hypoglycemia—associated admissions (*n*, %)	98, 12%	0.25

### Central nervous system

**Figure 1:**
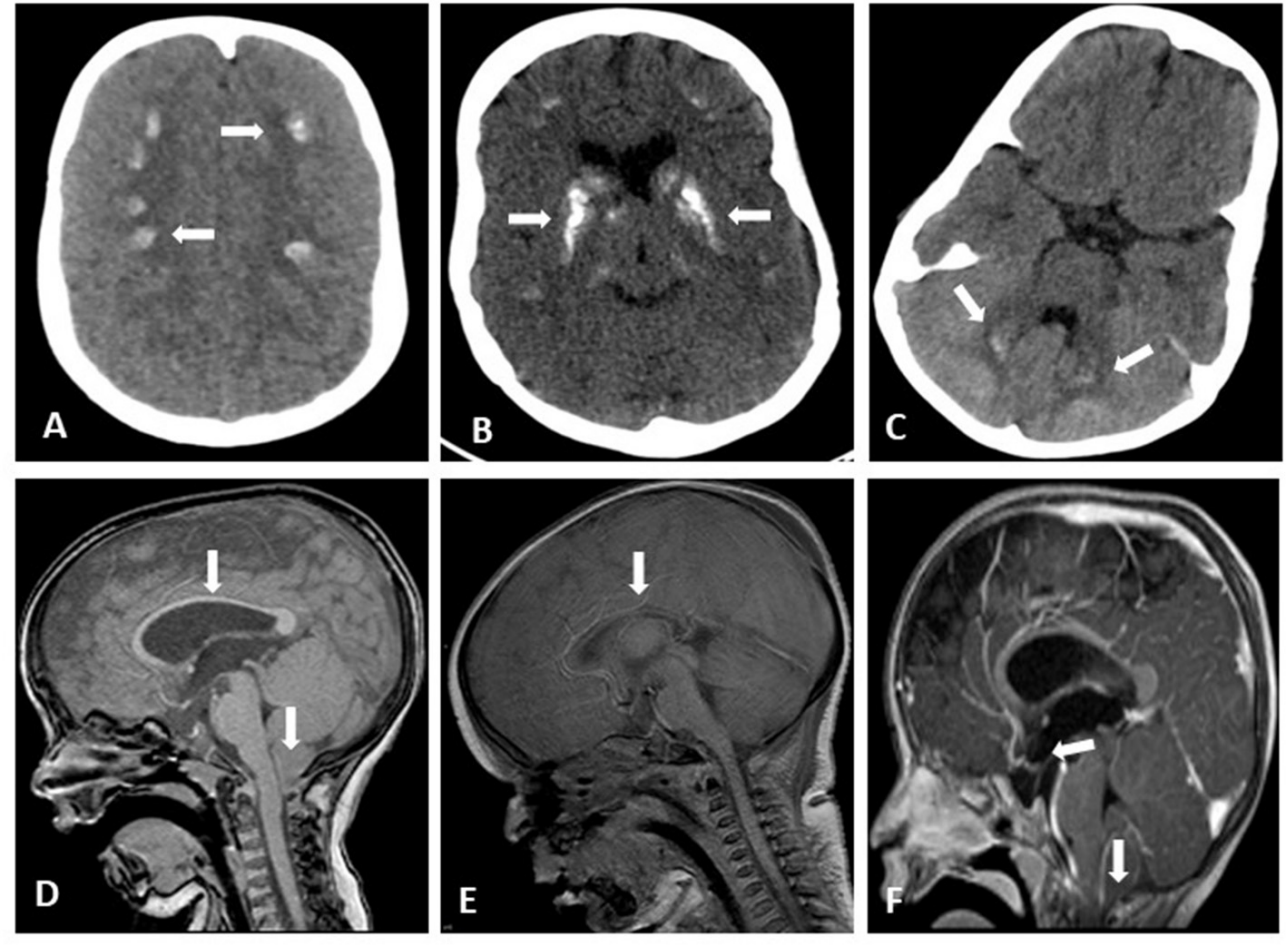
Brain imaging of HRD patients. Axial brain CT without contrast showing bilateral frontoparietal calcifications **(A)**, bilateral basal ganglia coarse calcifications **(B)** small bilateral Dentate Nucleus calcifications **(C)**; Sagittal brain MRI T1 without contrast showing Chiari Malformation and thin Corpus Callosum **(D)** considerable tightening of Corpus Callosum **(E)**; Sagittal brain MRI T1 with gadolinium showing drooping of cervical tonsils trough the foramen magnum and pituitary gland flattening **(F)**.

All patients had microcephaly. 39/63 (62%) of patients had had at least one seizure during their lifetime. The average age for the first event was 7 months (range: from first day of life to 47.5 months). All these patients had an EEG examination, and 18 of them (46%) had multiple pathologic patterns. Thirty-three patients underwent brain imaging (21 had a CT scan, 9 had both a CT scan and MRI, and 3 patients had an MRI), demonstrating brain anomalies in 90% of the patients examined including: basal ganglia calcifications found in 51% of examined patients, and other calcified areas included frontal subcortical areas and the dentate nucleus; 15% showed varying degrees of brain atrophy; 15% exhibited tightening of the corpus callosum; 9% had a Chiari type 1 malformation, 9% were hydrocephalic; septo-optic dysplasia and craniosynostosis were found in one patient, and partial agenesis of the corpus callosum was demonstrated in another patient. Note that some patients had more than one anomaly (Supplementary Table 1; Figure 1).

Median tibial and peroneal nerve motor and sensory nerve conduction studies performed in 11 children were within a normal range for age. Unfortunately, formal intellectual evaluation was not performed in most patients in our cohort. Nevertheless, all patients suffered from intellectual disability, with a significant delay in achieving developmental milestones. When achieved, walking was established between 2–4 years. Speech was never achieved by some patients, and others had incomprehensible speech.

### Ophthalmic complications

Twenty-five patients were referred to ophthalmologic evaluation due to abnormal physical examination findings. All demonstrated multiple eye anomalies involving all eye segments with 3/25 (12%) having more than one anomaly. Corneal anomalies were described in 4 patients (2 patients with opacities and 2 with micro cornea). Cataract was described in 5 patients. Overall, 6 patients had retinopathy and retinal detachment, and 4 other patients had optic nerve abnormalities including one patient with septo-optic dysplasia. Coloboma, nystagmus, and pseudotumor cerebri (after initiation of growth hormone treatment) each appeared in a single patient. Strabismus was described along with these ocular findings in some patients, and as a single phenomenon in 6 patients (Supplementary Table 1).

### ENT

Otitis media was the most common infectious etiology found during hospitalization, with 42/63 (66%) of patients suffering from at least one episode. Removal of foreign bodies from the ear canal and tympanoplasty were undertaken in 4 patients. Both conductive and senso-neural hearing loss was documented in 6 patients.

### Cardiac

Only minor structural malformations were found in 5 patients, including atrial septal defect (ASD), mild tricuspid regurgitation (TR), and mild aortic regurgitation (AR).

### Gastrointestinal involvement

Of the entire cohort, 13/63 (20%) suffered from constipation, and most of them required treatment with laxatives and enemas during hospitalization. Six of the 63 patients (9.5%) were diagnosed with bowel obstruction based on clinical and radiographic findings. Both “true” (intraluminal obstruction) and “pseudo” obstructions were reported (2 and 4, respectively). Two patients had colonic obstruction, two had small intestinal obstruction, and another two patients had both. The youngest patient was diagnosed at the age of 8 years, and the oldest patient at 26 years (Supplementary Table 1). Four patients underwent laparotomy; pathology reports from 3 patients indicated normal presence of myenteric plexuses. One patient had a hypercalcemic episode concomitant with pseudo-obstruction. Phosphor levels were inconsistent among the patients during obstructive episodes.

### Renal anomalies

Of the patients, 32/63 had had at least one renal imaging, 31 an abdominal ultrasound, and one patient a CT scan. Mild congenital anomalies of the kidney and urinary tract (CAKUT) were found in 4 patients: 3 with hydronephrosis and one with unilateral a multicystic dysplastic kidney; none were accompanied by nephrocalcinosis. Of the 32 patients who had undergone renal imaging, 15 (47%) showed sonographic evidence of medullary nephrocalcinosis or nephrolithiasis. The youngest patient was diagnosed at 10 months with multiple unilateral renal calculi, and the oldest patient was diagnosed at 20 years of age. Three patients (20%) had spontaneous recovery of nephrocalcinosis, and one patient required surgery. Nephrocalcinosis was associated with serious bacterial infections; 7 patients were diagnosed with sepsis (5) and urinary tract infection (2) due to *E. coli* spp. and *Klebsiella* spp., and nephrocalcinosis was found in 5 of 6 patients examined (ultrasound was not performed in one patient). Two patients demonstrated proteinuria, and one suffered from nephrogenic diabetes insipidus, attributed to nephrocalcinosis and tubular damage. Except during concurrent illness, creatinine and urea blood levels were within a low-to-normal range for ages in all patients.

### Musculoskeletal involvement

Four patients suffered from fractures, a single fracture for each patient. Three female patients had various degrees of kyphoscoliosis developed during the second decade of life.

### Calcium homeostasis

The target therapeutic calcium range was defined as slightly below or in the lower range of the prevailing reference interval that changed through the study period. Thus, significant hypocalcemia was diagnosed when calcium levels were <7.5 mg/dL. To avoid excessive representation of multiple tests performed during hospitalization periods, we analyzed average calcium at 2 weeks intervals. Significant hypocalcemia was observed in 63% of tests. Symptomatic hypocalcemia (including laryngospasm, wheezing, seizures, and ECG anomalies) was observed as early as a few hours after birth. It was also one of the most common causes of hospitalization among our patients, with an incidence of 1.21 per life years. Of the 63 patients, 20 (32%) were hospitalized due to hypercalcemia >10.8 mg/dL, at a mean age of 42 months (range: 1–86 months). Calcium phosphor product > 70 occurred in 11% of tests and did not correlate with the occurrence of nephrocalcinosis and nephrolithiasis.

## Discussion

In this study, we aimed to describe the multisystem involvement in HRD syndrome. Our prolonged follow-up of the largest reported cohort of patients allows us to shed light on under-reported features of HRD patients. These may be related to TBCE loss of function in multiple organs or to the disrupted calcium and phosphor homeostasis that, in turn, may be the first step in multiple cascades, culminating in seizures, bowel obstruction, nephrolithiasis, and susceptibility to infections.

Microtubules are a part of the cytoskeleton, with *TBCE* protein important for microtubule polymerization (3). While *TBCE* RNA can be detected in most human tissues, *TBCE* protein is found mainly in the cerebellum, testis, spleen, and thyroid. Lesser expression is found also in respiratory, gastrointestinal, and genitourinary systems (https://www.proteinatlas.org/ENSG00000285053-TBCE/tissue). The precise molecular mechanisms through which *TBCE* mutations cause hypoparathyroidism is still not clear, as the gene product is nearly absent in normal mature parathyroid tissue. Some mechanisms have been proposed and include a possible role of the protein in embryonic parathyroid cell migration, as well as other *TBCE* functions yet to be elucidated (10).

Hypocalcemia is the most prevalent complication seen among our patients; hypercalcemia is an iatrogenic complication, reflecting the everyday difficulties in balance severe hypoparathyroidism. Both hypercalcemia and vitamin D treatment are known risk factors for development of nephrocalcinosis. Thus, the therapeutic goal of calcium in patients with hypoparathyroidism is in the lower range to avoid aggressive calcium excretion and potentially stone formation. Calcium phosphor product is used to assess the risk for nephrolithiasis among patients with renal failure and adults with hypoparathyroidism (11). The introduction of a low solute diet to our cohort combined with target therapeutic calcium is probably the cause for why calcium phosphor product is not useful for predicting risk for nephrolithiasis. In our cohort, sonographic evidence for nephrocalcinosis and nephrolithiasis was found in 47% of patients as early as the first year of life, but mostly in the second decade of life. Elhassanien et al. reported renal calcification among 66% of 24 HRD patients (12). Levy et al. (13) reported a cohort of 29 pediatric patients with primary hypoparathyroidism (mainly due to Digeorge syndrome and idiopathic cases); nephrocalcinosis was observed in 38% of subjects. The most significant predictors for stone formation were degree of relative hypercalcemia, hyperphosphatemia, and lower glomerular filtration rate (GFR). While the calculus formation was progressive in most cases, it was reversible in 18% of patients and correlated with therapeutic range calcium. In our cohort, 20% of patients had reversible nephrolithiasis. It seems that lowering calcitriol treatment was an important feature of this phenomenon.

Bowel obstruction is a serious and life-threatening complication found among our cohort. Constipation was reported by 20% of patients and in 5/6 patients later diagnosed with bowel obstruction, probably representing the first step in this process. In one patient, bowel obstruction was the first presentation. In adults, the main gastrointestinal manifestation of hypoparathyroidism is steatorrhea (14); Christodoulou et al. (15) reported one HRD patient with bile salt-induced diarrhea and progressive respiratory failure, while endoscopy showed bilious reflux up to the esophagus with normal villous architecture. In our cohort, no patient had chronic diarrhea, but both intraluminal fecal impactions and “pseudo” obstructions were reported. Previously reported HRD cases included one patient with superior mesenteric artery syndrome causing bowel obstruction (16) and another patient with intestinal pseudo-obstruction due to visceral myopathy (17). Other motility problems (gastro-esophageal reflux disease, delayed gastric emptying demonstrated in a multiple upper GI series) were also reported in our cohort. Hypercalcemia impairs bowel motility; as smooth muscle contraction is mediated *via* calcium ions. Constipation and abdominal pain are common symptoms of hypercalcemia in children (18). Few reports in the literature regarding hypercalcemia-induced colonic and intestinal pseudo-obstructions can be found (19–21); however, all of them describe adults, and most related to hematological malignancies. All patients who suffered from bowel obstruction had episodes of hypercalcemia, but only a single episode was concomitant with bowel obstruction. Intestinal and colonic pseudo-obstructions are also known features of acute hyperphosphatemia, with most cases described as iatrogenic and related to oral and enema preparations. Except for one patient, hyperphosphatemia was not observed before or during obstructive episodes although it is a key feature of PTH deficiency. Histopathological findings of one patient with pseudo-obstruction demonstrated focal fibrosis, scattered Cajal cells, myocytes, and muscular layer atrophy in both the colon and ileum. Ganglion cells were present in all plexuses, and no nerve hypertrophy was seen. Pathology reports from two other patients operated on for colonic perforation demonstrated normal myenteric plexuses. These pathology findings are similar to a previous report by Pal et al. (17) suggesting visceral myopathy is the cause for intestinal pseudo-obstruction in HRD patients. As in our cohort, calcium homeostasis was unrelated to symptoms.

TBCE protein is expressed in the gastrointestinal tract and is important for neuron survival (22, 23). Thus, although normal plexuses were reported, their function may be impaired. Suggesting that both neurogenic and myopathic mechanisms, rather than PTH deficiency or electrolyte disturbances, are the likely causes for GI involvement in HRD.

The interplay between profound and often uncontrolled hypocalcemia to recurrent episodes of hypoglycemia is the main explanation for the high prevalence of convulsions in HRD. Seizures were reported in 93–100% of patients in previous cohorts (12, 24). The prevalence in our cohort is 62%, probably reflecting our center's extensive experience with early diagnosis and treatment soon after birth. Brain anomalies may also cause seizures. Three main anomalies were described in our cohort: calcifications, developmental brain anomalies (Chiari malformation and partial agenesis of the corpus callosum), and brain atrophy. Basal ganglia calcifications (sometimes referred to as Fahr's disease), a known complication of hypoparathyroidism, were demonstrated in 51% of our patients. A previous study reported brain calcifications in 38% of HRD patients (12). Tubulinopathies are a group of developmental brain malformations caused by mutations in genes encoding tubulins and their chaperones. Those include lissencephalies, cerebellar hypoplasia, and agenesis of the corpus callosum and cortical dysplasia as a part of migratory neuronal disorders. Indeed, structural abnormalities of the pituitary gland and corpus callosum have been reported in HRD (8, 12, 15, 25). Similar developmental brain and eye anomalies were present in our cohort, emphasizing the role of *TBCE* during embryonal development of the central nervous system. Brain atrophy was also demonstrated in our cohort. Tauopathies are a group of neurodegenerative diseases related to pathological formation of hyperphosphorylated tau protein insoluble aggregates known as neurofibrillary tangles. The most famous is Alzheimer's disease. TBCE-deficient mice (*pmn*/*pmn*) do not demonstrate hypoparathyroidism, but neurological abnormalities are present, as TBCE is important for axonal tubulin routing from the Golgi apparatus and, in its absence, axonal degeneration occurs (22). In 2016, Sferra et al. reported a biallelic c.464T>A (p.Ile155Asn) *TBCE* mutation causing early-onset progressive encephalopathy with distal spinal muscular atrophy (26). Similar to the *pmn*/*pmn* mouse, none of the 6 patients that were reported suffered from hypoparathyroidism, and MRI findings were consistent with cerebellar, corpus callosum, and brainstem atrophy. Optic atrophy was also reported. Recent work by Fujiwara et al. describes an miRNA-mediated knockdown system of TBCE-deficient mice, causing a reduction in properly folded tubulins and accumulation of phosphorylated tau in the cell bodies (23). Considering these findings, we preformed nerve conduction studies in our patients; all had normal results. More research is needed to clarify the role of TBCE and central and peripheral nervous system development and function.

HRD patients are susceptible to severe infections, *TBCE* RNA is found in multiple leukocyte lineages (https://www.proteinatlas.org/ENSG00000285053-TBCE/tissue). Although *TBCE* deficiency does not alter WBC number, it impairs phagocytosis and neutrophil migration (6). One major site for *TBCE* activity is the spleen; in fact, functional asplenia was reported as a major cause of HRD patient mortality (6). Asplenia is usually related to capsular bacterial infections (e.g., *Pneumococcus* spp. *Neisseria meningitidis*, and *Haemophilus influenza*). Indeed, amoxicillin prophylaxis was a pivotal point in our cohort survival as the under 5 years mortality rate declined dramatically between 1989–2000 and 2000–2019 from 77% of patients to 24% of patients. The under 5 years mortality rate in Israel during that time was 9.2 and 4.8 cases for 1,000 births, respectively, Mortality rate, under-5 (per 1,000 live births)—Israel|Data (worldbank.org). Thus, vaccinations and antibiotic prophylaxis should comprise the standard of care in patients with HRD. It seems that *Enterobacteriaceae* are also emerging pathogens among our patients and were associated with nephrocalcinosis. Other risk factor may be a shift in bacterial flora due to prophylactic antibiotic treatment and vaccinations as all episodes recorded after the year 2006.

In this report, we described multiple clinical features of HRD syndrome. The strength of our study is a large cohort followed in one medical center and allow us to demonstrate that unique complications reported earlier occurs in a statistically significant manner.

We acknowledge some limitations in our study: First, due its retrospective design, we were unable to estimate the precise prevalence of each condition. Second, although distinctive dysmorphic features are the hallmark of HRD, genetic diagnosis was available in only 40% of our patients. Thirteen years have passed since the first patient included in this cohort was born until publication of the *TBCE* mutation (3). During that time, many patients succumbed to infections during early childhood and, thus, genetic analysis was unavailable. Many of these patients originated from families in which *TBCE* molecular diagnosis was later established. Third, we were unable to assess if some of the complications were related to the disease or to the course of treatment.

In summary, HRD is a rare disease with many manifestations. The prolonged study period and the substantial experience of our center allowed us to observe multiple complications including the high prevalence of central nervous system developmental anomalies, nephrolithiasis, and risk for *Enterobacteriaceae* infections and bowel obstructions. Active surveillance and treatment are indicated for these more common complications (Table 2).

**Table 2:** Recommendations for HRD patients follow up and treatment.

**Infectious and immune system:**
- Prophylactic antibiotic treatment with amoxicillin
- Prompt antibiotic treatment during episodes of febrile illness, including gram negative bacterial coverage, especially when urinary tract abnormalities are present
- Anti-Pneumococcal vaccines
**Endocrine and metabolic:**
- Calcium supplements and active vitamin D analogs to achieve calcium levels slightly below or in the lower range of the reference interval
- Annual screening for hypothyroidism and adrenal insufficiency
**Gastrointestinal and nutrition:**
- A low solute and phosphor diet (e.g., breast milk and SIMILAC 60/40)
- Close follow-up of bowel habits and prompt treatment of constipation
**Renal:**
- Annual ultrasound to screen for nephrocalcinosis starting at age 1 year
**Vision:**
- Ophthalmic evaluation during the first months of life to assess treatable vision Impairments (e.g., cataracts) and further follow-up as needed
**Hearing:**
- An annual hearing screen during childhood to assess both conductive (due to recurrent otitis media) and sensory-neural hearing loss

## Data availability statement

The original contributions presented in the study are included in the article/supplementary material, further inquiries can be directed to the corresponding author/s.

## Ethics statement

The studies involving human participants were reviewed and approved by Soroka Medical Center. Written informed consent from the participants' legal guardian/next of kin was not required to participate in this study in accordance with the national legislation and the institutional requirements.

## Author contributions

OD and EH conceptualized this work and wrote the first draft of the manuscript. OD and RA collected the data. RN, DS, DW, LC, ME-S, OB, GL, RS, NL, and AH contributed to the areas relevant to their expertise and edited the manuscript. All authors were involved in preparation of the manuscript, contributed to the article, and approved the submitted version.

## Conflict of interest

The authors declare that the research was conducted in the absence of any commercial or financial relationships that could be construed as a potential conflict of interest.

## Publisher's note

All claims expressed in this article are solely those of the authors and do not necessarily represent those of their affiliated organizations, or those of the publisher, the editors and the reviewers. Any product that may be evaluated in this article, or claim that may be made by its manufacturer, is not guaranteed or endorsed by the publisher.

## Supplementary material

Supplementary Table 1 Clinical phenotype of 63 HRD patients.
