# Supplementary material for: Hypoparathyroidism-retardation-dysmorphism syndrome—Clinical insights from a large longitudinal cohort in a single medical center
Source: Front Pediatr. 2022 Jul 22;10:916679. doi: 10.3389/fped.2022.916679 (PMC9352926; doi:10.3389/fped.2022.916679)
Supplement: Supplementary Table 1 — Clinical phenotype of 63 HRD patients. [file Table_1.DOCX]

**Table 1 supplementary:**

| **patient #** | Demographics | | Brain | | | Eye | Renal | | GI tract |
| --- | --- | --- | --- | --- | --- | --- | --- | --- | --- |
|  | Gender | survival | Imaging | Findings | Seizures |  | last US Findings (age in years) | reversible?  (age at last US) | constipation/obstruction (age at first event) |
| 1 | F | no | N/A | N/A | yes | N/A | N/A | N/A | constipation |
| 2 | M | no | N/A | N/A | yes | N/A | N/A | N/A | ─ |
| 3 | F | no | CT | normal | no | N/A | N/A | N/A | ─ |
| 4 | F | no | CT | BGC | yes | N/A | MNC (10) | no (21) | pseudo - SBO + CO (26) |
| 5 | F | no | N/A | N/A | no | N/A | N/A | N/A | constipation |
| 6 | M | no | CT | normal | yes | coloboma, RD | N/A | N/A | ─ |
| 7 | F | no | N/A | N/A | yes | N/A | dysplastic kidney (1) | ─ | ─ |
| 8 | F | no | CT | BGC, BA | yes | N/A | CT - normal (22) | ─ | CO (12) |
| 9 | M | no | CT | hydrocephalus | yes | N/A | nephrolitheasis (20) ‡ | ─ | constipation |
| 10 | F | yes | CT+MRI | BGC | yes | cataract | MNC (5) | no (19) | constipation |
| 11 | M | no | N/A | N/A | no | N/A | N/A | N/A | ─ |
| 12 | F | no | MRI | BGC, hippocampal atrophy | yes | N/A | nephrolitheasis (13) ‡ | yes (17) | pseudo - SBO (17) |
| 13 | M | no | CT + MRI | Rt. microphthalmia | yes | RD | nephrolitheasis (9) ‡ | no (14) | ─ |
| 14 | M | no | N/A | N/A | yes | R | MNC (6) | ─ | ─ |
| 15 | M | no | CT | BGC | yes | N/A | N/A | ─ | ─ |

| **patient #** | Demographics | | Brain | | | Eye | Renal | | GI tract |
| --- | --- | --- | --- | --- | --- | --- | --- | --- | --- |
|  | Gender | survival | Imaging | Findings | Seizures |  | last US Findings (age in years) | reversible?  (age of last US) | constipation/obstruction (age at first event) |
| 16 | M | no | N/A | N/A | yes | N/A | MNC (1) | no (2) | ─ |
| 17 | F | no | N/A | N/A | no | cataract | N/A | N/A | ─ |
| 18 | F | no | N/A | N/A | yes | cataract | nephrolitheasis (7) ‡ | ─ | constipation |
| 19 | F | no | N/A | N/A | yes | N/A | nephrolitheasis (6) | ─ | SBO + CO (8) |
| 20 | F | no | CT | BGC | no | N/A | normal (6 months) | ─ | ─ |
| 21 | M | no | CT | paCC | no | nystagmus | N/A | N/A | ─ |
| 22 | M | no | N/A | N/A | no | strabismus | hydronephrosis (1 month) | ─ | ─ |
| 23 | F | no | N/A | N/A | yes | N/A | N/A | N/A | ─ |
| 24 | M | no | CT + MRI | BGC | yes | pseudo tumor cerebri | normal (4) ‡ | ─ | ─ |
| 25 | F | no | N/A | N/A | yes | N/A | normal (1) | ─ | ─ |
| 26 | F | yes | CT | BGC | yes | N/A | N/A | N/A | constipation |
| 27 | M | yes | CT | BGC | yes | Mi, ONA | N/A | N/A | pseudo CO (16) |
| 28 | F | no | N/A | N/A | no | corneal opacities | normal (1 month) | ─ | ─ |
| 29 | F | yes | CT | BGC | yes | strabismus | MNC (5) | no (14) | ─ |
| 30 | F | no | CT | hydrocephalus | no | N/A | N/A | N/A | ─ |
| 31 | M | no | N/A | N/A | no | N/A | N/A | N/A | ─ |

| **patient #** | Demographics | | Brain | | | Eye | Renal | | GI tract |
| --- | --- | --- | --- | --- | --- | --- | --- | --- | --- |
|  | Gender | survival | Imaging | Findings | Seizures |  | last US Findings (age in years) | reversible?  (age at last US) | constipation/obstruction (age at first event) |
| 32 | F | no | CT | BA | yes | N/A | N/A | N/A | ─ |
| 33 | F | yes | CT+MRI | BGC and BA | yes | R | MNC (11) | N/A | pseudo - SBO (9) |
| 34 | F | no | CT | BGC | yes | Mi,R | N/A | N/A | ─ |
| 35 | F | no | N/A | N/A | no | N/A | N/A | N/A | ─ |
| 36 | M | yes | CT + MRI | CM, tCC | yes | N/A | nephrolithiasis (1) | yes - operative (12) | ─ |
| 37 | M | yes | CT | BGC | yes | ONA | N/A | N/A | ─ |
| 38 | F | yes | CT | BGC | yes | N/A | N/A | N/A | ─ |
| 39 | F | no | MRI | C, SOD, tCC | yes | optic atrophy | N/A | N/A | ─ |
| 40 | F | yes | CT + MRI | tCC and BA | yes | cataract | MNC (8) ‡ | no (9) | ─ |
| 41 | F | yes | N/A | N/A | yes | strabismus | nephrolithiasis (3) | yes (4) > MNC (8) | ─ |
| 42 | M | no | CT | hydrocephalus | no | corneal opacities | N/A | N/A | ─ |
| 43 | F | yes | N/A | N/A | yes | strabismus | normal (8) ‡ | ─ | ─ |
| 44 | F | yes | MRI | CM, tCC | yes | N/A | normal (8) | ─ | ─ |
| 45 | M | no | N/A | N/A | no | N/A | N/A | N/A | ─ |
| 46 | M | yes | N/A | N/A | yes | N/A | normal (8) | ─ | ─ |
| 47 | F | yes | CT | BGC and CM | yes | strabismus | normal (9) | ─ | ─ |
| 48 | F | yes | N/A | N/A | yes | ONA | normal (8) | ─ | ─ |

| **patient #** | Demographics | | Brain | | | Eye | Renal | | GI tract |
| --- | --- | --- | --- | --- | --- | --- | --- | --- | --- |
|  | Gender | survival | Imaging | Findings | Seizures |  | last US Findings (age in years) | reversible? (age of last US) | constipation/obstruction (age at first event) |
| 49 | F | yes | CT + MRI | normal | yes | strabismus | normal (6) ‡ | ─ | ─ |
| 50 | F | no | CT + MRI | tCC | no | RD | N/A | N/A | ─ |
| 51 | F | yes | N/A | N/A | no | N/A | N/A | N/A | ─ |
| 52 | M | yes | CT | BGC | no | N/A | normal (3) | ─ | ─ |
| 53 | M | yes | N/A | N/A | yes | N/A | N/A | N/A | ─ |
| 54 | M | yes | CT | BGC | yes | N/A | hydronephrosis (1) | ─ | ─ |
| 55 | M | yes | N/A | N/A | no | N/A | N/A | N/A | ─ |
| 56 | M | yes | N/A | N/A | yes | N/A | normal (1 month) | ─ | ─ |
| 57 | F | yes | N/A | N/A | no | N/A | N/A | N/A | constipation |
| 58 | F | yes | N/A | N/A | no | N/A | Nephrolithiasis  (10 months) | Yes  (16 months) | ─ |
| 59 | F | yes | CT + MRI | brain ischemia | no | N/A | N/A | N/A | constipation |
| 60 | M | yes | N/A | N/A | no | cataract | N/A | N/A | ─ |
| 61 | F | yes | N/A | N/A | no | N/A | N/A | N/A | ─ |
| 62 | M | yes | CT | tCC | no | N/A | N/A | N/A | ─ |
| 63 | F | yes | N/A | N/A | no | N/A | hydronephrosis (1 month) | ─ | ─ |

BCG - basal ganglia calcifications, BA - brain atrophy, paCC - partial agenesis of corpus callosum, CM - Chiari malformation, tCC - tightening of corpus callosum, C - craniosynostosis, SOD - septo-optic-dysplasia, RD - retinal detachment, R - retinopathy, ONA - optic nerve anomaly, Mi - microcornea, MNC - medullary nephrocalcinosis, SBO - small bowel obstruction, CO - colonic obstruction, ‡ small kidneys
